# Contemporary Regional Disparities in Robotic‐Assisted Rectal Resection in Japan: A Nationwide Population‐Based Study Using the National Database Open Data

**DOI:** 10.1002/ags3.70220

**Published:** 2026-04-06

**Authors:** Ryo Ohta, Takeshi Yamada, Key Uehara, Hiromichi Sonoda, Akihisa Matsuda, Seiichi Shinji, Yasuyuki Yokoyama, Goro Takahashi, Nobuhiko Taniai, Hiroshi Yoshida

**Affiliations:** ^1^ Department of Gastroenterological Surgery Nippon Medical School Musashikosugi Hospital Kawasaki Japan; ^2^ Department of Gastroenterological Surgery Nippon Medical School Tokyo Japan

**Keywords:** National Database Open Data in Japan, population‐based study, regional disparity, robotic‐assisted rectal resection, urban–rural difference

## Abstract

**Background:**

Robotic‐assisted rectal resection has expanded in Japan since national health insurance reimbursement began in 2018; however, its population‐level regional distribution remains unclear. We evaluated temporal trends and contemporary regional disparities using the National Database (NDB) Open Data.

**Methods:**

We analyzed NDB Open Data for fiscal years 2014–2023. All patients undergoing rectal resection, irrespective of disease etiology, were included. Procedures were classified as open, laparoscopic, or robotic‐assisted. We assessed temporal trends and age‐ and sex‐stratified surgical volumes. For 2023, prefecture‐level surgical rates per 100 000 population and robotic proportions were calculated. Standardized claim ratios (SCRs) were computed to adjust for age and sex. Population density was used as a proxy for urbanization. Prefectures were categorized into urban and rural groups using the highest and lowest population‐density quartiles (middle two quartiles excluded), and robotic proportions were compared using the Mann–Whitney *U* test as an exploratory analysis.

**Results:**

Robotic‐assisted rectal resections increased markedly after 2018, whereas open surgery declined. Substantial inter‐prefectural variation was observed in surgical volume and robotic utilization, and heterogeneity persisted after age‐ and sex‐standardization using SCRs. Urban prefectures exhibited a higher median robotic proportion than rural prefectures (29.3% vs. 19.0%), although this difference did not reach statistical significance (*p* = 0.09). Treating population density as a continuous measure, robotic proportion was positively associated with population density (Spearman *ρ* = 0.338, *p* = 0.020).

**Conclusions:**

Robotic‐assisted rectal resection expanded in Japan after reimbursement, yet marked inter‐prefectural heterogeneity remained, underscoring the need for continued nationwide monitoring of geographic variation in adoption.

## Graphical abstract

Robotic‐assisted rectal resection in Japan increased markedly after national insurance reimbursement in 2018, with a concurrent decline in open surgery. Using NDB Open Data, we found substantial inter‐prefectural heterogeneity in surgical volume and robotic utilization that persisted after age and sex standardization (SCR). Urban–rural differences were exploratory, while population density showed a modest positive association with robotic utilization, suggesting that resource concentration may shape diffusion patterns.

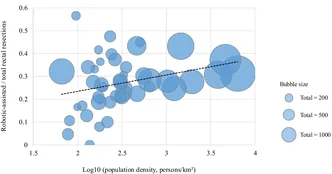

## 1 Introduction

Robotic‐assisted rectal resection has rapidly expanded in Japan since its inclusion in the national health insurance system in 2018. Technical advantages such as articulated multi‐joint instruments, high‐definition three‐dimensional visualization, and magnified operative views have improved surgical precision. Recent studies have reported not only a reduction in local recurrence but also improvements in disease‐free survival and postoperative urinary function in patients undergoing robotic‐assisted rectal resection, underscoring its growing clinical value [1, 2].

In the Japanese context, advanced surgical platforms and high‐volume tertiary centers are typically concentrated in more densely populated prefectures. Accordingly, we used prefecture‐level population density as a pragmatic proxy for urbanization and healthcare‐resource concentration, and treated the urban–rural comparison as exploratory rather than a definitive classification of access.

Despite these advantages, the dissemination of robotic surgery is constrained by substantial capital investment and high annual maintenance costs. As a result, access to robotic‐assisted rectal resection may vary substantially among medical institutions, raising concerns regarding geographic inequities in the delivery of advanced surgical care in Japan.

Several nationwide studies have examined regional differences in robotic‐assisted rectal resection, using large‐scale databases, including the National Clinical Database (NCD), the National Database of Health Insurance Claims and Specific Health Checkups of Japan (NDB), and inpatient administrative databases [3, 4, 5]. However, most of these investigations included periods prior to insurance reimbursement or were conducted during the early phase of robotic surgery adoption, when its penetration was limited.

Therefore, the present study aimed to comprehensively evaluate contemporary regional disparities in robotic‐assisted rectal resection in Japan using the most recent National Database Open Data, which captures healthcare utilization for nearly the entire population under the universal health insurance system.

## 2 Methods

### 2.1 Data Source

We obtained nationwide aggregated data on rectal resection surgery from the National Database (NDB) Open Data released by the Ministry of Health, Labour and Welfare of Japan. The NDB contains claims data covering more than 95% of all reimbursed medical services under the universal health insurance system. Annual data from fiscal year 2014 through 2023 were extracted for analysis. Because the NDB Open Data are fully anonymized and publicly available, this study did not require institutional review board approval or informed consent, in accordance with the Ethical Guidelines for Medical and Health Research Involving Human Subjects in Japan.

### 2.2 Study Population

Patients who underwent rectal resection surgery between fiscal years 2014 and 2023 were included, regardless of underlying disease etiology. Cases were identified using procedure‐specific reimbursement codes corresponding to rectal resection surgery. Patients with missing prefecture information were excluded from the regional analyses.

### 2.3 Definitions of Surgical Procedures

Rectal resection surgeries were classified into three categories according to reimbursement codes:

- Open surgery
- Laparoscopic surgery
- Robotic‐assisted surgery

Annual numbers and proportions of each surgical approach were calculated. Age‐ and sex‐stratified analyses were performed for the year 2023.

### 2.4 Regional Classification and Population Adjustment

Prefecture‐level population data were obtained from the Statistics Bureau of Japan. Surgical rates were calculated as the number of rectal resection surgeries per 100 000 population for each prefecture.

The robotic surgery proportion was defined as the number of robotic‐assisted rectal resections divided by the total number of rectal resections in each prefecture. Standardized claim ratios (SCRs) were calculated for robotic‐assisted rectal resection to evaluate inter‐prefectural variation after adjusting for age and sex (national average = 1.0). For each prefecture, the expected number of robotic‐assisted rectal resections (E) was computed by applying the national age‐ and sex‐specific robotic utilization rates to the corresponding prefecture populations and summing across strata; SCR was defined as observed/expected (O/E). Ninety‐five percent confidence intervals (CIs) for SCRs were calculated assuming a Poisson distribution for the observed counts (exact method). Because prefectures with small expected counts may yield imprecise SCR estimates, we interpreted estimates for such prefectures with caution.

For the urban–rural comparison (exploratory), prefectures were ranked by population density and classified using quartiles: those in the highest quartile were defined as urban (*n* = 12) and those in the lowest quartile as rural (*n* = 12). The middle two quartiles were excluded from this two‐group comparison to maximize contrast in population density. Given the potential arbitrariness of any single cut‐point, we emphasized this analysis as hypothesis‐generating and interpreted results cautiously.

### 2.5 Statistical Analysis

Continuous variables were expressed as medians with interquartile ranges. Differences in robotic surgery rates between urban and rural prefectures were compared using the Mann–Whitney *U* test. As sensitivity analyses, we assessed the association between population density (continuous, log‐transformed) and prefecture‐level robotic surgery proportion using Spearman's rank correlation across all 47 prefectures. We also fitted a binomial generalized linear model with a logit link, modeling the number of robotic‐assisted procedures as events and total rectal resections as trials, with the natural logarithm of population density (In‐transformed) as the predictor. The odds ratio corresponding to a doubling of population density was calculated as exp.(βx In2), where β represents the regression coefficient for In(population density). The association between population density and prefecture‐level robotic surgery proportion was visualized using a bubble plot, with bubble size proportional to the total number of rectal resections. All statistical analyses were two‐sided, and a *p* value < 0.05 was considered statistically significant. Statistical analyses were performed using SPSS Statistics version 27 (IBM Corp., Armonk, NY, USA).

## 3 Results

### 3.1 Temporal Trends of Surgical Approaches

**FIGURE 1:**
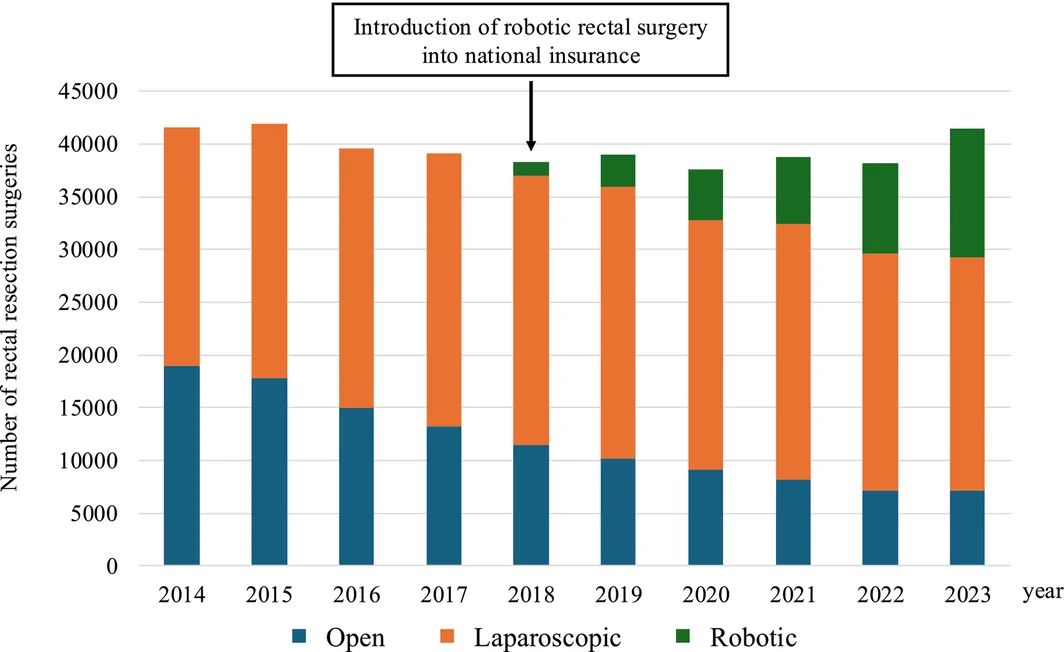
Annual trends in the number of rectal resection surgeries in Japan from 2014 to 2023, stratified by surgical approach (open, laparoscopic, and robotic‐assisted), based on the National Database Open Data. The annotation indicates the timing of national insurance reimbursement for robotic‐assisted rectal resection (2018).

The annual number of rectal resection surgeries according to surgical approach from 2014 to 2023 is shown in Figure 1. During the study period, the total number of rectal resection surgeries gradually increased. Open surgery demonstrated a continuous decline throughout the observation period, whereas laparoscopic surgery remained relatively stable. In contrast, the number of robotic‐assisted rectal resections markedly increased after 2018, coinciding with the introduction of national health insurance reimbursement for robotic surgery. By 2023, robotic‐assisted procedures accounted for a substantial proportion of all rectal resections, suggesting a substantial expansion in adoption at the national level.

### 3.2 Age‐ and Sex‐Stratified Surgical Volume

**FIGURE 2:**
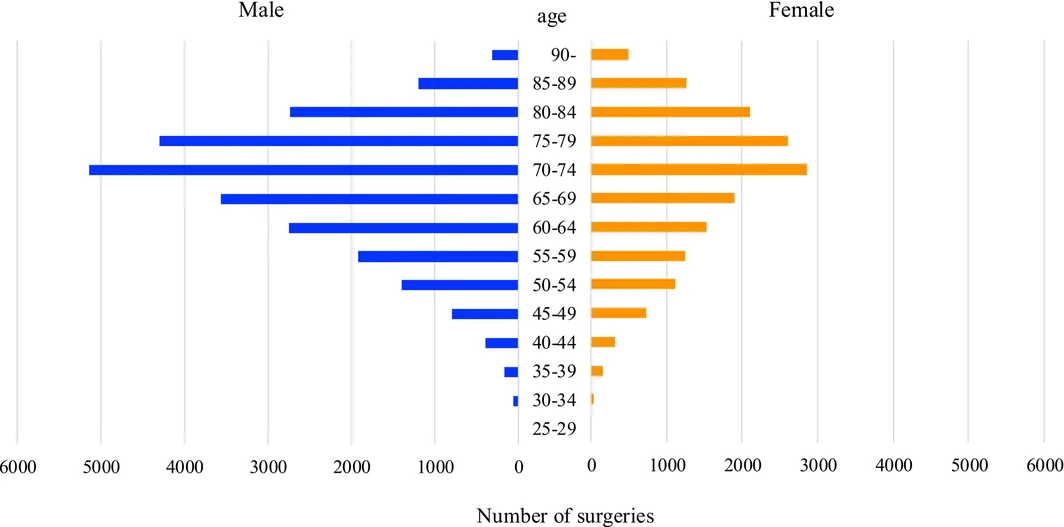
Total number of rectal resections stratified by age group and sex from 2014 to 2023. Bars represent annual procedure counts within each age stratum for female (left) and male (right) patients.

The total number of rectal resections increased steadily from 2014 to 2023 in both sexes. Male patients consistently accounted for a larger proportion of surgeries compared with female patients throughout the study period (Figure 2).

### 3.3 Age‐Stratified Surgical Approach in 2023

**FIGURE 3:**
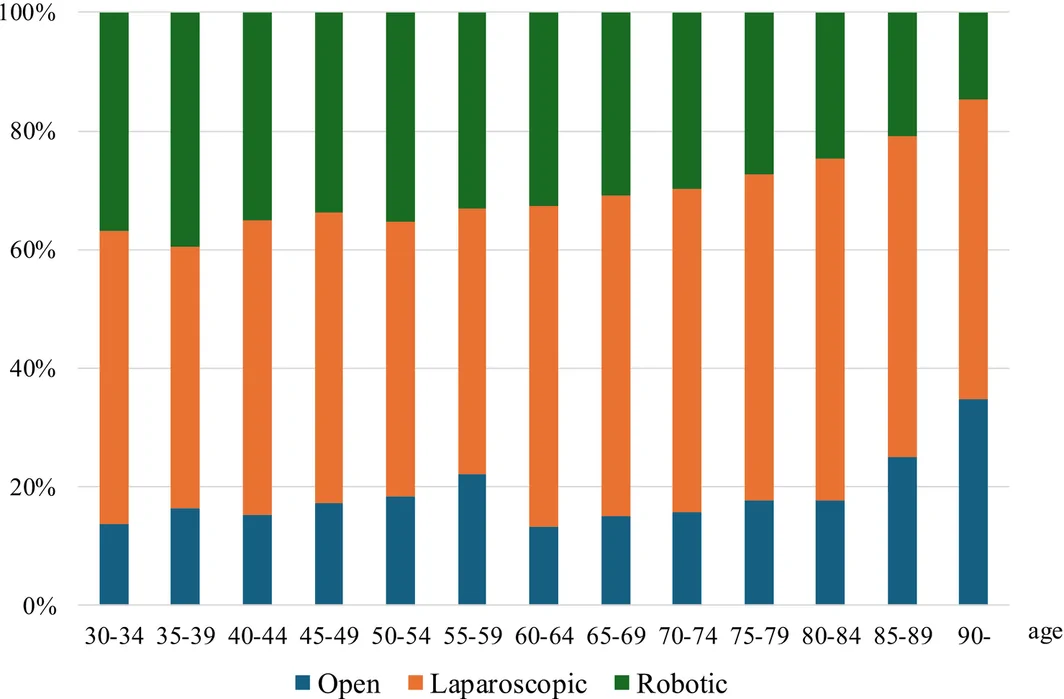
Age‐stratified distribution of surgical approaches for rectal resection in 2023. Stacked bars show the proportion of open, laparoscopic, and robotic‐assisted procedures within each 5‐year age group.

In 2023, laparoscopic surgery remained the dominant surgical approach across all age groups. The proportion of robotic‐assisted surgery was highest among patients aged 35–39 years and gradually declined in older age groups (Figure 3).

### 3.4 Geographic Distribution of Surgical Volume and Robotic Proportion

**FIGURE 4:**
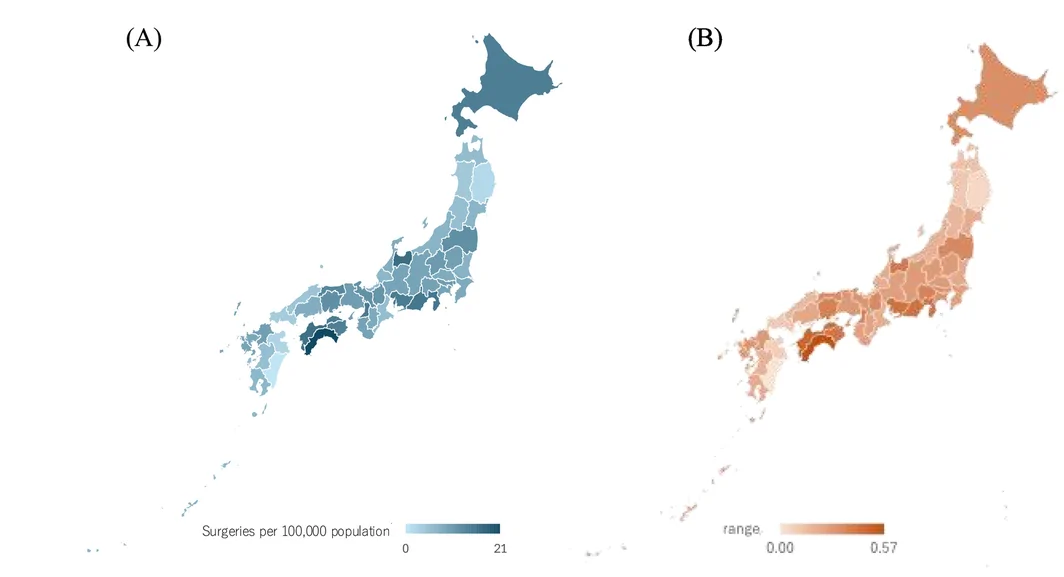
(A) Prefecture‐level rectal resection rate per 100 000 population in 2023, calculated as the number of rectal resections divided by the prefecture population and multiplied by 100 000. (B) Geographic distribution of the proportion of robotic‐assisted rectal resection in 2023, calculated as robotic‐assisted rectal resections divided by total rectal resections in each prefecture. Prefecture boundaries are shown for reference.

Marked geographic variation was observed in the rectal resection rate per 100 000 population across prefectures in 2023 (Figure 4A). In addition, the proportion of robotic‐assisted rectal resection varied substantially by region, with higher utilization appearing in several metropolitan prefectures (Figure 4B).

### 3.5 SCR‐Based Inter‐Prefectural Comparison

**FIGURE 5:**
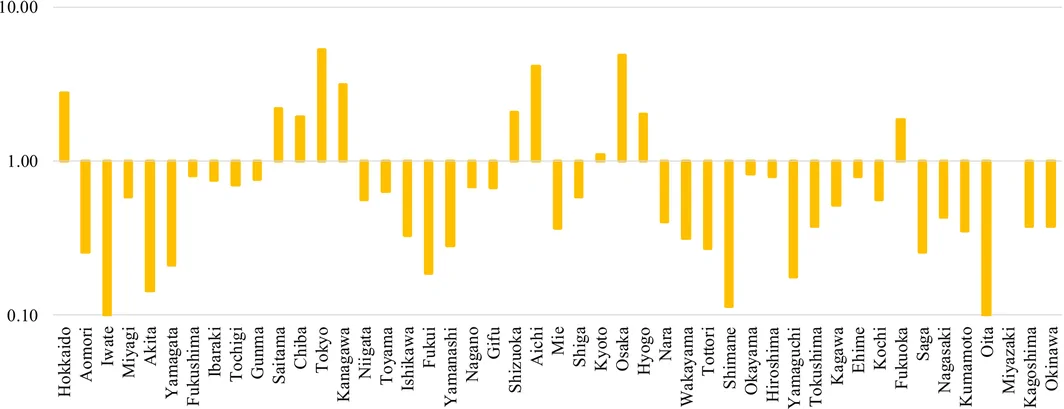
Standardized claim ratio (SCR) of robotic‐assisted rectal resection across prefectures in 2023 after adjustment for age and sex (national average = 1.0). SCR is defined as the observed number of robotic‐assisted rectal resections divided by the expected number derived from national age‐ and sex‐specific rates applied to prefecture populations; values are plotted on a logarithmic scale.

The SCR analysis demonstrated wide inter‐prefectural variation in the utilization of robotic‐assisted rectal resection after adjustment for age and sex. Several urban prefectures showed SCRs well above the national average, whereas multiple rural prefectures demonstrated markedly low SCRs, suggesting residual inter‐prefectural heterogeneity (Figure 5).

### 3.6 Urban–Rural Disparity Analysis

**FIGURE 6:**
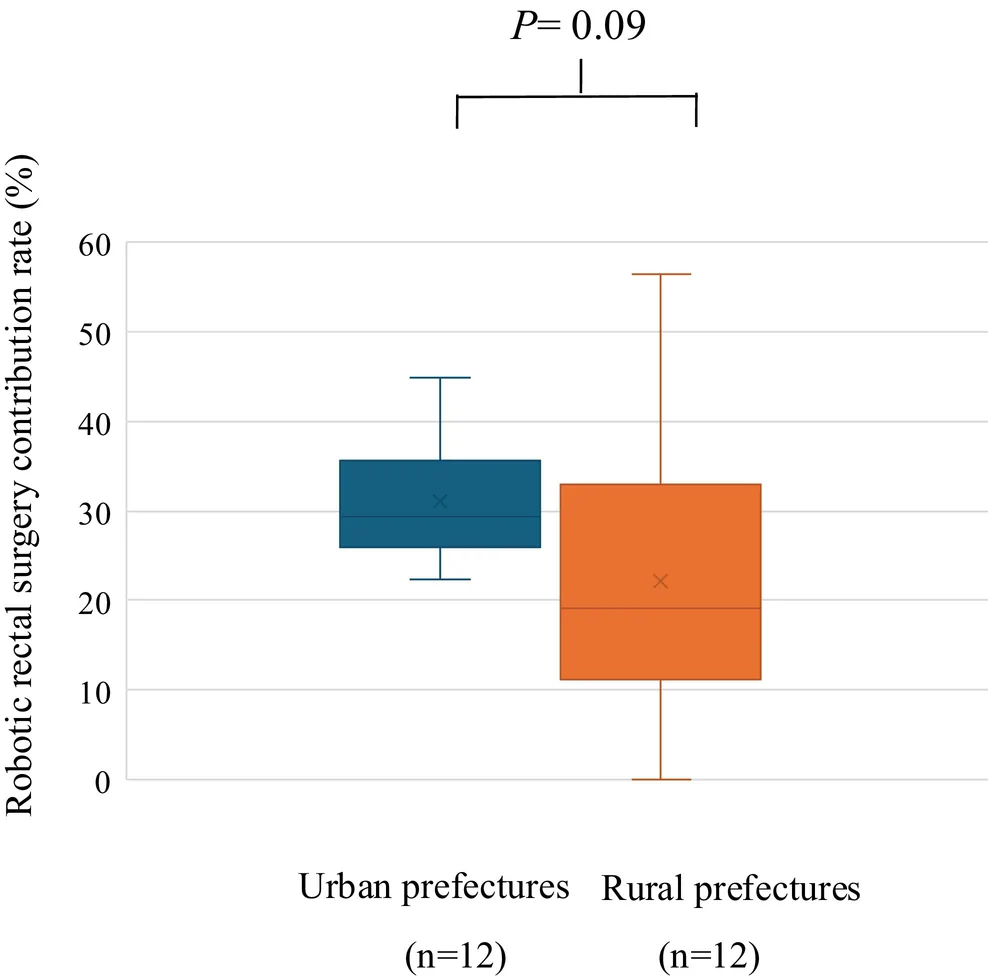
Comparison of the proportion of robotic‐assisted rectal resection between urban and rural prefectures classified by population‐density quartiles (urban: highest quartile; rural: lowest quartile; *n* = 12 each; middle two quartiles excluded). The *p* value was calculated using the Mann–Whitney *U* test (two‐sided).

**FIGURE 7:**
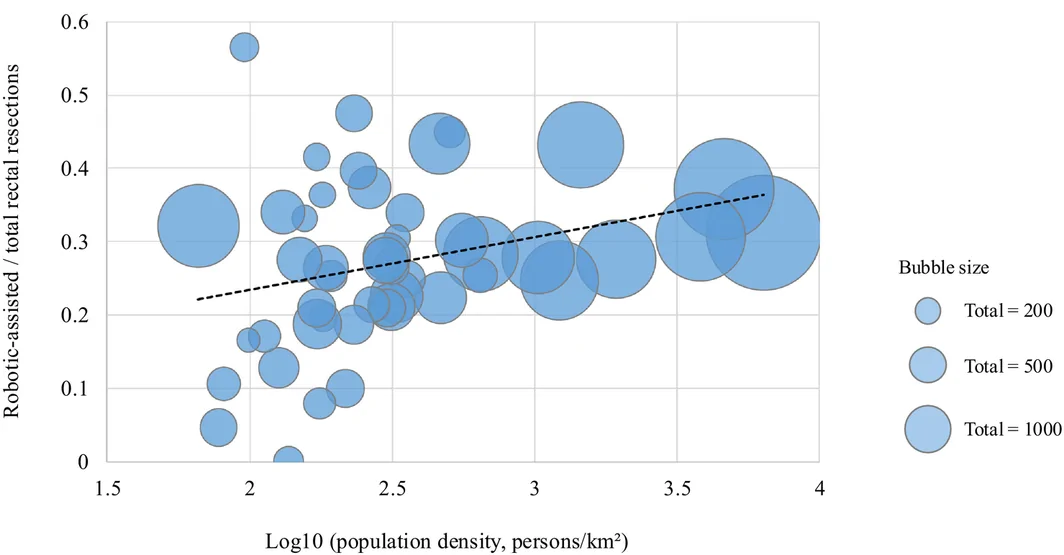
Association between population density and the proportion of robotic‐assisted rectal resection across 47 prefectures (fiscal year 2023). Each point represents a prefecture; the x‐axis shows population density on a log10 scale (persons/km^2^), and the y‐axis shows the proportion of robotic‐assisted procedures among all rectal resections. Bubble size is proportional to the total number of rectal resections. The dashed line represents a simple linear fit for visualization.

Prefectures classified as urban tended to have a higher proportion of robotic‐assisted rectal resection than rural prefectures; however, the difference did not reach statistical significance (median 29.3% vs. 19.0%, *p* = 0.09, Mann–Whitney *U* test; Figure 6). Treating population density as a continuous measure, the prefecture‐level robotic proportion was positively associated with population density (Spearman *ρ* = 0.338, *p* = 0.020; Figure 7). In a binomial GLM sensitivity analysis using the natural logarithm of population density, higher population density was associated with a higher robotic proportion (regression coefficient for In (population density) 0.100, 95% CI 0.085–0.115; *p* < 0.001). This corresponds to an odds ratio of 1.07 (95% CI 1.06–1.08) for a doubling of population density, calculated as exp. (β x In2).

## 4 Discussion

This nationwide population‐based study using the National Database Open Data yielded three major findings. First, the adoption of robotic‐assisted rectal resection in Japan has increased rapidly since its inclusion in the national health insurance system in 2018, accompanied by a gradual decline in open surgery. Second, substantial inter‐prefectural variation was observed in both the surgical volume and the proportion of robotic‐assisted procedures, even after standardization for age and sex using SCRs. Finally, robotic‐assisted utilization tended to be higher in urban than rural prefectures and was positively associated with population density; however, these findings should be interpreted as exploratory rather than definitive evidence of inequity.

The marked increase in robotic‐assisted rectal resection observed after 2018 is consistent with evidence from randomized controlled trials and meta‐analyses demonstrating favorable perioperative and oncological outcomes of robotic surgery for rectal cancer. The ROLARR trial showed a lower conversion rate in robotic‐assisted surgery compared with laparoscopic surgery, particularly in technically challenging cases such as male and obese patients [6]. Subsequent randomized and observational studies further reported comparable or improved oncological outcomes and postoperative functional recovery following robotic total mesorectal excision [7, 8, 9, 10, 11, 12]. In China, the REAL trial demonstrated improved short‐term outcomes and postoperative urinary function in patients undergoing robotic rectal surgery [1, 2]. These accumulating data likely facilitated institutional adoption once reimbursement became available, highlighting the critical role of policy‐level financial coverage in shaping national surgical practice patterns.

Despite the overall diffusion of robotic surgery, we identified pronounced inter‐prefectural variation in both the absolute surgical volume and the proportion of robotic‐assisted rectal resections, even after adjustment for age and sex. Similar geographic and socioeconomic disparities in robotic colorectal surgery have been reported in the United States and Europe, where hospital volume, institutional resources, and surgeon availability were identified as major determinants of robotic adoption [13, 14, 15, 16]. Japanese nationwide database studies have likewise demonstrated substantial heterogeneity in minimally invasive surgery utilization across regions [3]. Our SCR‐based approach further revealed latent disparities that were not apparent from crude surgical counts alone, underscoring the importance of population‐standardized metrics when evaluating regional equity in access to advanced surgical technologies.

In the urban–rural comparison, urban prefectures exhibited a higher median proportion of robotic‐assisted rectal resection than rural prefectures; however, this difference did not reach statistical significance (*p* = 0.09) and should therefore be considered hypothesis‐generating rather than definitive evidence of inequity. Given the operational nature of the quartile‐based classification and the limited statistical power of a dichotomous comparison at the prefectural level, we additionally evaluated population density as a continuous measure to reduce reliance on an arbitrary threshold. In this analysis, population density was positively associated with robotic utilization at the prefecture level (Spearman ρ = 0.338, *p* = 0.020), with consistent results in a binomial GLM sensitivity analysis. These findings align with reports from other healthcare systems suggesting that diffusion of robotic surgery follows a stepwise pattern, initially concentrated in high‐volume metropolitan centers before gradually disseminating to regional hospitals [14, 16, 17, 18]. According to diffusion‐of‐innovation theory, early adopters are typically resource‐rich institutions, whereas broader penetration occurs only after sufficient technical expertise, infrastructure, and economic feasibility are established [19, 20]. Collectively, our results are compatible with the possibility that resource concentration may contribute to heterogeneous diffusion; however, the aggregated nature of prefecture‐level data does not allow us to distinguish between limited access and appropriate centralization to high‐volume centers. Accordingly, continued surveillance is warranted to determine whether these emerging gradients translate into clinically meaningful disparities over time. Because population density is a proxy for multiple structural factors‐including facility distribution, system availability, and referral patterns‐our findings should primarily be interpreted as reflecting geographic variation in the concentration of robotic surgery rather than definitive evidence of inequitable access.

The financial burden associated with robotic platforms remains a substantial barrier to widespread dissemination. Previous cost analyses have demonstrated significantly higher acquisition and maintenance costs for robotic systems compared with conventional laparoscopic equipment [5, 21, 22, 23]. These economic constraints disproportionately affect smaller and rural hospitals, potentially reinforcing structural disparities in access to robotic surgery. From a health policy perspective, it is therefore essential to balance innovation with equity, particularly in a universal healthcare system such as that of Japan. The National Database provides a unique opportunity to monitor the population‐level impact of reimbursement policies on healthcare delivery and regional equity [24, 25].

Several limitations warrant consideration. First, the NDB Open Data do not allow direct differentiation between a true lack of access and appropriate centralization of complex rectal surgery to high‐volume centers; therefore, geographic variation in utilization should not be interpreted solely as inequitable access. Second, the NDB Open Data are aggregated and claims‐based, precluding adjustment for important clinical variables such as indication (malignant vs. benign), tumor stage, neoadjuvant therapy, or long‐term oncological outcomes. Because diagnosis information is not available in the Open Data, our analysis necessarily included all rectal resections irrespective of etiology; results may not perfectly reflect patterns specific to rectal cancer. Third, hospital‐level factors, including institutional volume, surgeon experience, and robotic system availability, could not be evaluated. Fourth, analyses were conducted at the prefectural level, and intraprefectural heterogeneity (e.g., urban–rural differences within large prefectures) could not be assessed. Finally, potential misclassification of procedure codes cannot be completely excluded.

In conclusion, using nationwide population‐based data from the NDB Open Data, we demonstrated an increase in robotic‐assisted rectal resection in Japan following insurance reimbursement, with substantial inter‐prefectural variation in utilization. These findings should be interpreted as reflecting geographic variation in the concentration of robotic surgery rather than definitive evidence of inequitable access. Continuous nationwide monitoring is warranted to ensure equitable access to advanced surgical technologies across regions.

## Author Contributions

Study conception and design: Ryo Ohta, Takeshi Yamada, Nobuhiko Taniai, and Hiroshi Yoshida. Data acquisition: Ryo Ohta, Key Uehara, Hiromichi Sonoda, Seiichi Shinji, Yasuyuki Yokoyama, Goro Takahashi, and Takeshi Yamada. Statistical analysis: Ryo Ohta and Akihisa Matsuda. Interpretation of data: Ryo Ohta, Takeshi Yamada, Nobuhiko Taniai, and Hiroshi Yoshida. Drafting of the manuscript: Ryo Ohta, Takeshi Yamada, Nobuhiko Taniai, and Hiroshi Yoshida. Critical revision of the manuscript: All authors. All authors approved the final manuscript.

## Funding

The authors have nothing to report.

## Disclosure

Registry and the Registration No. of the Study/Trial: N/A.

Animal Studies: N/A.

## Ethics Statement

This study used the National Database Open Data, which are fully anonymized and publicly available. Accordingly, institutional review board approval and informed consent were not required.

## Consent

The authors have nothing to report.

## Conflicts of Interest

Dr. Kay Uehara is a current Editorial Board Member of Annals of Gastroenterological Surgery. She was not involved in the editorial evaluation or decision‐making process for this manuscript. The other authors declare no Conflicts of Interest for this article.

## Data Availability

The data that support the findings of this study are available in the National Database (NDB) Open Data in Japan at https://www.mhlw.go.jp/stf/seisakunitsuite/bunya/0000177182.html. These data were derived from the following resources available in the public domain: ‐ Ministry of Health, Labour and Welfare. Government of Japan, https://www.mhlw.go.jp/index.html.
